# Compensatory proximal malalignment despite preserved macroscopic gait in pediatric flexible flatfoot: A dual-level analysis of static alignment and dynamic kinematics

**DOI:** 10.1371/journal.pone.0358183

**Published:** 2026-09-16

**Authors:** Haojie Wang, Mengxiang Li, Xiaomeng Liang, Weijian Wang, Pengren Luo, Jiaji Zhang, Yuxuan Du, Tan Liu, Boyu Zhang, Xu Wang, Zhefeng Jin, Yilin Zhu, Tao Han

**Affiliations:** 1 The Third Department of Sports Medicine, Wangjing Hospital, China Academy of Chinese Medical Sciences, Beijing, China; 2 Rehabilitation Division, Wangjing Hospital, China Academy of Chinese Medical Sciences, Beijing, China; 3 Department of Gynecology, Wangjing Hospital, China Academy of Chinese Medical Sciences, Beijing, China; Somaiya Vidyavihar University K J Somaiya College of Engineering, INDIA

## Abstract

**Background & objective:**

Previous biomechanical studies on pediatric flexible flatfoot have treated the subject as a single analytical unit, thus failing to elucidate mechanical transmission within the lower extremity closed kinetic chain. This study investigates the impact of distal arch collapse on macroscopic gait parameters, static joint alignment, and dynamic kinematic deviation by integrating a dual-level analytical strategy at both the subject and individual limb levels.

**Methods:**

153 children with pediatric flexible flatfoot (25 unilateral, 128 bilateral) were included, and 3D gait data from 306 lower extremities were analyzed. At the subject level, macroscopic spatiotemporal parameters were compared using the Mann-Whitney U test. At the limb level, linear mixed-effects models (LMMs) compared static 3D joint angles and Gait Variable Scores (GVS) between normal feet and flatfeet, treating subject as a random effect and adjusting for age, body mass index (BMI), and unilateral leg length.

**Results:**

At both the subject and limb levels, no significant between-group differences were observed in any core spatiotemporal parameters (e.g., Mean Velocity, Frequency, and Step Width) (P > 0.05). Regarding static joint alignment at the limb level, the static Hip Rotation angle on the flatfoot side exhibited significantly less external rotation (i.e., relative internal rotation) compared to the normal side (−2.11° ± 0.67° vs. −5.79° ± 1.79°, *P* = 0.043). During dynamic walking, the Hip Abduction/Adduction GVS on the flatfoot side was significantly elevated (*P* = 0.032). However, comprehensive gait indices, including the GDI and GPS, showed no significant overall between-group differences.

**Conclusion:**

Pediatric flexible flatfoot was associated with subtle proximal biomechanical alterations despite preserved macroscopic gait characteristics. Changes in static hip alignment and dynamic frontal-plane hip kinematics may therefore provide complementary information beyond conventional gait measures, supporting a broader kinetic-chain assessment in clinical evaluation and follow-up.

## Introduction

Pediatric flexible flatfoot (FFT) is a common musculoskeletal disorder of the lower extremities. It is primarily characterized by the collapse of the medial longitudinal arch and excessive subtalar joint eversion [1,2]. These structural abnormalities significantly alter the mechanical characteristics of the lower extremities. Although many children remain asymptomatic in the short term, clinical observations indicate that persistent structural deformities can lead to altered stress distribution, lower extremity fatigue, and an elevated risk of injury [3–5] Current evidence suggests that the biomechanical consequences of FFT involve complex structural and sensorimotor adaptations that extend well beyond local foot and ankle complex mechanics [6]. Specifically, within the closed kinetic chain of the lower extremities, distal instability is transmitted proximally, thereby altering the kinematics and dynamic motor control of proximal joints [7–9].

According to biomechanical principles, static alignment significantly influences dynamic function [10] Clinically, adaptive changes within the closed kinetic chain of the lower extremities often manifest as altered neuromuscular recruitment patterns [11]and an increased demand on proximal stabilizing muscle groups to maintain posture and distal control [12]. These muscle groups must counteract the abnormal joint moments (e.g., excessive or misdirected inversion/eversion and rotational moments) generated by the flatfoot deformity to prevent further biomechanical dysfunction.Although these functional compensations persist to maintain overall walking efficiency, this motor strategy—rooted in an initial structural malalignment—may induce an occult loss of postural stability. Furthermore, prolonged and unmanaged proximal compensation can lead to adverse consequences, including accelerated joint wear [13], patellofemoral pain syndrome [14], and altered force transmission across the articular surfaces of the hip and knee [15].

However, research concerning the biomechanical consequences of FFT—particularly regarding its systematic impact on overall postural control and proximal joints of the lower extremities—remains relatively limited. First, previous studies have predominantly focused on joint angle alterations during dynamic gait cycles, frequently overlooking potential three-dimensional (3D) kinematic abnormalities during static stance. Consequently, few studies have systematically explored how static 3D joint malalignments translate into dynamic gait deviations. Second, when evaluating dynamic gait quality, isolated spatiotemporal parameters often mask multi-planar kinematic deficits [16]. The incorporation of objective, comprehensive gait indices—such as the Gait Profile Score (GPS) and Gait Variable Scores (GVS)—can more sensitively quantify neuromuscular adaptability to distal deformities [17]. More importantly, regarding research design and statistical methodology, prior studies have largely utilized the subject as a single analytical unit. This approach overlooks the fact that the closed kinetic chain of the lower extremities is fundamentally a limb-specific mechanical transmission process. Clinically, FFT presents with either unilateral or bilateral involvement; grouping solely by subject may obscure critical biomechanical differences between an unaffected limb and a collapsed arch within the same patient. Furthermore, the uncontrolled pooling of bilateral limb data violates the assumption of statistical independence. Finally, because pediatric gait is significantly influenced by growth and development, studies failing to strictly control for confounding factors, such as age and body mass index (BMI), often yield inconsistent and less robust conclusions [18].

To address these limitations, this study employed a high-precision infrared optoelectronic motion capture system and a dual-level analytical strategy integrating both the limb and subject levels. By utilizing linear mixed-effects models (LMMs) to rigorously control for within-subject correlations and developmental covariates, we compared the biomechanical characteristics of normal feet and flatfeet, treating the individual lower extremity as the primary unit of analysis. Specifically, this study aims to investigate the following two primary objectives: (1) Assessing static differences: To quantitatively compare the 3D spatial alignment of the lower extremity joints between the two groups during static stance; (2) Quantifying dynamic function and gait deviations: To determine whether static structural alterations influence the spatiotemporal parameters of dynamic walking, and to reveal underlying multi-planar kinematic deviations using comprehensive gait indices (i.e., GDI, GPS, and GVS).

## Materials and methods

### Research design

This single-center, retrospective observational study was conducted using data from the clinical gait database of the Third Department of Sports Medicine, Wangjing Hospital, China Academy of Chinese Medical Sciences, spanning the period from 2025 to 2026.All research data included in this study were accessed on 01/02/2026 from the clinical gait database.

### Ethical approval and informed consent

This study protocol was approved by the Ethics Committee of Wangjing Hospital, China Academy of Chinese Medical Sciences (Approval No.: WJEC-KT-2025–064-P001). Given the retrospective nature of this study, which involved no additional interventions or risks to the subjects, the requirement for informed consent was waived by the Ethics Committee. All data were strictly anonymized prior to analysis; personal identifying information (e.g., names and medical record numbers) was removed and replaced with unique identification codes. The collection, storage, and analysis of all data were conducted in strict accordance with the ethical principles outlined in the Declaration of Helsinki.

### Participants

#### Inclusion criteria.

(1) Patients who underwent 3D gait analysis and arch index measurement at our institution between January and December 2025;(2) A confirmed clinical diagnosis of FFT, established via physical examination and plantar pressure analysis (diagnostic reference standard: arch index > 0.28) [19];(3) Aged between 6 and 18 years;(4) Availability of complete arch index and 3D gait analysis data, without missing key parameters.

#### Exclusion criteria.

(1) Presence of concomitant neurological or neuromuscular disorders (e.g., cerebral palsy, poliomyelitis, peripheral neuropathy, or muscular diseases);(2) A history of lower extremity fracture, dislocation, joint replacement, or severe musculoskeletal deformity;(3) Diagnosis of inflammatory or degenerative joint diseases (e.g., rheumatoid arthritis, ankylosing spondylitis, or osteoarthritis);(4) Receipt of lower extremity surgery, orthotic interventions (bracing), or standardized rehabilitation within the 6 months prior to assessment;(5) Presence of lower extremity pain, limited mobility, or any other condition affecting natural gait during the assessment.

#### Grouping and sample size.

Based on the arch index and the laterality of the deformity, patients were categorized into a unilateral flatfoot group (n = 25) and a bilateral flatfoot group (n = 128). This yielded a total of 281 flatfeet and 25 normal feet for limb-level analysis. The sample size was determined by the number of eligible cases available during the study period; no a priori power analysis was conducted.

### Data collection

Arch Index Measurement: The BENECOR plantar pressure assessment system (Beijing Zhengxing Medical Technology Co., Ltd., Beijing, China) was utilized to simultaneously collect plantar pressure and morphological data. The arch index was calculated automatically, with a value > 0.28 serving as the diagnostic threshold for FFT.

Gait Parameter Acquisition: Three-dimensional gait data were acquired using an 8-camera infrared motion capture system (SMART-D 400, BTS Bioengineering, Milan, Italy) at a sampling rate of 100 Hz, integrated with synchronized force platforms sampling at 1000 Hz.

### Testing protocol

All assessments were administered by two uniformly trained clinicians. Testing was conducted in a standardized gait laboratory equipped with an 8-meter walkway, under controlled environmental conditions (i.e., level surface, ambient temperature, and absence of intense light interference).

Initially, basic anthropometric indices (height, weight, and BMI) and specific lower extremity parameters (including bilateral leg length, pelvic width, and pelvic depth) were recorded. All measurements were performed in duplicate, and the mean values were utilized for anthropometric scaling in the subsequent biomechanical model to normalize individual differences. Subsequently, plantar pressure distribution was recorded to calculate the bilateral arch index. Subjects stood barefoot in the center of the force platform with their arms resting naturally at their sides. The system synchronously acquired the plantar pressure data; this procedure was repeated twice to obtain an average value.

Following arch assessment, 22 retroreflective markers were attached to specific anatomical landmarks using hypoallergenic double-sided tape, in accordance with the Helen Hayes marker set [20,21]. These landmarks included the vertebra prominens (C7), bilateral acromion processes, bilateral anterior superior iliac spines (ASIS), midpoint of the posterior superior iliac spines (PSIS), and bilaterally on the greater trochanters, lateral and medial femoral condyles, fibular heads, lateral and medial malleoli, between the first and second metatarsal heads, and the calcaneus. For static calibration, subjects stood barefoot in the center of the capture volume in a neutral stance with their arms abducted horizontally for 5 seconds. This allowed for the spatial calibration of both the 3D infrared motion capture system and the force platforms. Post-calibration, the medial malleolus and medial femoral condyle markers were removed bilaterally to prevent marker collision and interference during dynamic walking trials. Subjects were then instructed to walk barefoot along the 8-meter walkway at a self-selected, comfortable pace. Following a period of acclimatization to the testing environment, each subject performed three walking trials, and for each trial a minimum of two valid, complete gait cycles were acquired. The data were then averaged across the three trials for statistical analysis to minimize intra-individual variability and ensure data reliability.

The following specific biomechanical indices were extracted for analysis: (1) Spatiotemporal parameters: Stride Time, Stance Time, Swing Time, Stance Phase, Swing Phase, Single Support Phase, Double Support Phase, Mean Velocity, Frequency, Stride Length, Step Length, and Step Width; (2) Static joint angle parameters: Pelvis Tilt, Pelvis Obliquity, Pelvis Rotation, Hip Flexion/Extension, Hip Abduction/Adduction, Hip Rotation, Knee Flexion/Extension, Ankle Dorsi/Plantarflexion, and Foot Progression Angle; (3) Comprehensive gait indices: The Gait Deviation Index (GDI) was utilized to assess overall gait pathology; the Gait Profile Score (GPS) was employed to evaluate gait asymmetry and quantify the overall deviation of kinematic variables. Furthermore, the Gait Variable Score (GVS), which decomposes the GPS across nine specific kinematic variables, was used to evaluate the deviation of individual joint kinematics.

### Statistical analysis

All statistical analyses were performed using SPSS software (version 26.0; IBM Corp., Armonk, NY, USA). The Shapiro-Wilk test was utilized to assess the normality of continuous variables. Normally distributed continuous variables are presented as mean ± standard deviation (SD), whereas non-normally distributed data are expressed as median (interquartile range, IQR). To evaluate local mechanical transmission within the closed kinetic chain of the lower extremities, the individual limb was selected as the unit of analysis for static 3D joint angles, spatiotemporal parameters, and comprehensive gait indices (GDI, GPS, and GVS). Given the nested structure of the data (i.e., the inherent correlation between bilateral limbs within the same subject), linear mixed-effects models (LMMs) were employed to compare parameter differences between normal feet and flatfeet with medial longitudinal arch collapse. In these models, the diagnosis of FFT (arch index > 0.28) was designated as a fixed effect, while subject ID was included as a random intercept to account for within-subject bilateral non-independence. To rigorously control for the confounding effects of pediatric growth and development on joint kinematics and gait, age, body mass index (BMI), and unilateral leg length were included as covariates. A compound symmetry covariance structure was specified for the models. The LMM results are reported as estimated marginal means (EMMs) with standard errors (SEs). A P-value < 0.05 was considered statistically significant.

## Results

### Baseline characteristics

A total of 153 subjects were included in this study, comprising 25 in the unilateral flatfoot group and 128 in the bilateral flatfoot group. All statistical tests for baseline comparisons were two-tailed. No significant between-group differences were observed regarding age (*Z* = −0.206, *P =* 0.837), height (*Z =* −0.417, *P =* 0.677), weight (*Z* = −0.111, *P =* 0.912), or BMI (*Z* = −0.600, *P =* 0.549). Additionally, gender distribution did not differ significantly between the groups (*χ*2 = 0.041, *P =* 0.839). Overall, the baseline demographic and anthropometric characteristics of the two groups were highly comparable (Table 1).

**Table 1:** Demographic characteristics of the participants.

Characteristics	UFFT (N = 25)	BFFT(N = 128)	*Z/χ2*	*P*
Age(year)	12.00 (11.00, 14.00)	12.00 (10.00, 15.00)	*Z* = −0.206	0.837
Height(m)	162.00 (151.50, 170.00)	160.00 (150.00, 170.75)	*Z* = −0.417	0.677
Weight(kg)	49.00 (41.50, 61.75)	50.00 (39.63, 65.00)	*Z* = −0.111	0.912
BMI (kg/m^2^)	18.25 (16.42, 22.95)	20.05 (16.74, 23.34)	*Z* = −0.600	0.549
Male/female	15 (60.0%)/ 10 (40.0%)	74 (57.8%)/ 54 (42.2%)	*χ*² = 0.041	0.839

UFFT: Unilateral flexible flatfoot; BFFT: Bilateral flexible flatfoot; m: meter; kg: kilogram.

### Global spatiotemporal parameters at the subject level

Regarding macroscopic dynamic function, this study compared the overall walking performance of patients with unilateral versus bilateral involvement, utilizing the subject as the analytical unit. Since the data were not normally distributed, a nonparametric Mann-Whitney U test was employed to compare the unilateral and bilateral flatfoot subgroups. The results indicated that overall walking efficiency was highly consistent between children with unilateral and bilateral flatfoot. During natural walking, no statistically significant between-group differences were observed in Mean Velocity (*Z* = −0.384, *P =* 0.701), Frequency (*Z* = −0.400, *P =* 0.689), or Step Width (*Z* = −0.812, *P =* 0.417). This suggests that bilateral, as opposed to unilateral, structural deformities do not lead to a significant deterioration of macroscopic spatiotemporal parameters during dynamic walking (Table 2, S1 Fig).

**Table 2:** Comparison of gait parameters between unilateral flatfoot and bilateral flatfoot.

Parameters	UFFT (N = 25)	BFFT (N = 128)	Z	*P*
Mean Velocity(m/s)	1.00 (1.00, 1.10)	1.00 (0.90, 1.10)	−0.384	0.701
Frequency(steps/min)	108.00(99.60,114.60)	107.40(100.95,115.65)	−0.400	0.689
Step Width(m)	0.11 (0.08 0.14)	0.11 (0.09, 0.14)	−0.812	0.417

UFFT: Unilateral flexible flatfoot; BFFT: Bilateral flexible flatfoot; steps/min: steps per minute; m/s: meter per second.

S1 Fig shows the comparison results of mean velocity, stride frequency, and step width in sequence using violin plots, with no statistically significant differences.

### Spatiotemporal parameters at the limb level

At the limb level, the individual limb was utilized as the analytical unit to compare gait performance between normal feet and flatfeet, following adjustment for relevant covariates using linear mixed-effects models (LMMs). No statistically significant differences were observed in any spatiotemporal parameters across temporal, spatial, or gait cycle phase proportions (P > 0.05). This indicates that during level walking, the flatfoot deformity does not directly alter the temporal rhythm or spatial span of the ipsilateral limb within the fundamental gait cycle. Consequently, the macroscopic spatiotemporal execution capacity of the affected lower extremity remains preserved (Table 3, S2 Fig).

**Table 3:** Comparison of spatio-temporal parameters between normal foot and flat foot.

Parameters	Normal(N = 25)	FFT (N = 281)	Mean Diff (95% CI)	*P*
Stride Time (s)	1.14 ± 0.03	1.13 ± 0.01	0.01 (−0.04, 0.06)	0.614
Stance Time (s)	0.71 ± 0.02	0.71 ± 0.01	0.01 (−0.02, 0.04)	0.602
Swing Time (s)	0.43 ± 0.02	0.43 ± 0.01	0.00 (−0.04, 0.04)	0.989
Stance Phase (%)	62.07 ± 1.59	62.30 ± 0.54	−0.23 (−3.45, 2.99)	0.889
Swing Phase (%)	37.95 ± 1.32	37.74 ± 0.48	0.21 (−2.45, 2.87)	0.876
Single Support Phase (%)	36.40 ± 1.46	37.96 ± 0.48	−1.56 (−4.52, 1.40)	0.301
Double Support Phase (%)	13.16 ± 2.53	15.00 ± 0.73	−1.84 (−7.06, 3.38)	0.489
Stride Length (m)	1.14 ± 0.02	1.14 ± 0.01	0.00 (−0.04, 0.05)	0.844
Step Length (m)	0.58 ± 0.01	0.57 ± 0.01	0.01 (−0.01, 0.04)	0.261

FFT: flexible flatfoot.s: second; m: meter; %: percentage.

S2 Fig shows the comparison results of temporal parameters, spatial parameters, and gait cycle in sequence using bar charts, with no statistically significant differences.

### Static joint alignment at the limb level

During static stance, the three-dimensional (3D) kinematic characteristics of the lower extremity joints were compared between the normal and flatfoot sides. After adjustment via linear mixed-effects models (LMMs), both groups exhibited an external rotation posture in the transverse plane (with internal rotation defined as positive and external rotation as negative). Specifically, the static Hip Rotation angle on the flatfoot side (−2.11° ± 0.67°) demonstrated significantly less external rotation than that on the normal side (−5.79° ± 1.79°), representing a statistically significant between-group difference (*P =* 0.043). No significant differences were observed between the two groups in the static joint angles of the hip, knee, or ankle in other planes (P > 0.05; Table 4, S3 Fig).

**Table 4:** Comparison of static joint angles between normal foot and flat foot.

Parameters	Normal(N = 25)	FFT (N = 281)	Mean Diff (95% CI)	*P*
Pelvis Tilt(°)	12.01 ± 1.16	11.71 ± 0.69	0.30 (−1.71, 2.32)	0.767
Pelvis Obliquity(°)	0.59 ± 0.57	−0.05 ± 0.17	0.64 (−0.54, 1.81)	0.285
Pelvis Rotation(°)	0.25 ± 1.30	−0.02 ± 0.38	0.28 (−2.39, 2.95)	0.838
Hip Flexion/Extension(°)	6.09 ± 1.14	6.48 ± 0.88	−0.39 (−1.95, 1.17)	0.62
Hip Abduction/Adduction(°)	−3.94 ± 0.88	−3.18 ± 0.24	−0.76 (−2.57, 1.05)	0.411
Hip Rotation(°)	−5.79 ± 1.79	−2.11 ± 0.67	−3.68 (−7.25, −0.12)	**0.043** ^*^
Knee Flexion/Extension(°)	1.29 ± 0.93	1.83 ± 0.49	−0.54 (−2.26, 1.18)	0.535
Ankle Dorsi/Plantarflexion(°)	4.40 ± 1.35	2.80 ± 0.60	1.60 (−1.01, 4.21)	0.228
Foot Progression Angle(°)	−14.29 ± 1.88	−13.65 ± 0.62	−0.64 (−4.46, 3.17)	0.74

FFT: flexible flatfoot; °: degree.

S3 Fig shows the comparison results of static joint angles using bar charts, and significant differences were found in Hip Rotation angle (*P* < 0.05).

### Gait deviation and variability analysis

LMM analysis revealed no significant differences in comprehensive gait indices (i.e., GDI and GPS) between the unilateral and bilateral involvement groups (P > 0.05). However, the Hip Abduction/Adduction GVS was significantly higher on the flatfoot side (5.40° ± 0.14°) compared to the normal side (4.45° ± 0.43°; *P =* 0.032). No other significant between-group differences were observed in the GVS for kinematic variables across the hip, knee, or ankle joints (P > 0.05; Table 5, S4 Fig).

**Table 5:** Comparison of gait variability between normal foot and flat foot.

Parameters	Normal(N = 25)	FFT (N = 281)	Mean Diff (95% CI)	*P*
GDI(Sc)	86.02 ± 1.54	85.91 ± 0.71	0.11 (−2.83, 3.05)	0.943
GPS(°)	10.37 ± 0.44	10.55 ± 0.21	−0.18 (−1.02, 0.67)	0.679
Pelvis Tilt GVS(°)	6.70 ± 0.49	6.80 ± 0.46	−0.10 (−0.47, 0.27)	0.588
Pelvis Obliquity GVS(°)	3.63 ± 0.37	3.52 ± 0.14	0.12 (−0.62, 0.85)	0.754
Pelvis Rotation GVS(°)	4.96 ± 0.39	4.74 ± 0.18	0.22 (−0.54, 0.98)	0.567
Hip Flexion/Extension GVS(°)	13.06 ± 0.90	13.56 ± 0.58	−0.50 (−2.01, 1.00)	0.509
Hip Abduction/Adduction GVS(°)	4.45 ± 0.43	5.40 ± 0.14	−0.95 (−1.82, −0.08)	**0.032** ^*^
Hip Rotation GVS(°)	10.99 ± 1.02	12.31 ± 0.33	−1.32 (−3.41, 0.76)	0.213
Knee Flexion/Extension GVS(°)	15.13 ± 1.05	14.97 ± 0.38	0.17 (−1.94, 2.27)	0.877
Ankle Dorsi/Plantarflexion GVS(°)	11.42 ± 0.98	10.98 ± 0.37	0.44 (−1.51, 2.39)	0.654
Foot Progression GVS(°)	9.74 ± 0.81	8.51 ± 0.29	1.22 (−0.40, 2.85)	0.14

FFT: flexible flatfoot.; GDI: Gait Deviation Index; GPS: Gait Profile Score; GVS: Gait Variable Score; °: degree; Sc: score.

S4 Fig shows the comparison results of GPS, GDI, and GVS of each joint. Significant differences were observed in Hip Abduction/Adduction GVS (P < 0.05).

## Discussion

This study employed a dual-level analytical strategy integrating subject- and limb-specific data to characterize the relationship between pediatric FFT and lower-extremity biomechanics while controlling for developmental covariates. The principal finding was not a generalized impairment of walking performance, but rather a dissociation between preserved macroscopic gait characteristics and selective proximal kinematic alterations. Major spatiotemporal parameters remained comparable between groups and between limbs, whereas relative hip internal rotation during static stance and increased Hip Abduction/Adduction GVS during walking were identified on the affected side. This distinction is clinically relevant because apparently normal walking speed, cadence, and stride characteristics may coexist with more localized changes in lower-extremity alignment and movement.

All core spatiotemporal parameters, including Mean Velocity, Frequency, Stride Length, and gait-cycle phase proportions, showed no significant differences (P > 0.05) at either the subject or limb level. Rather than indicating an absence of biomechanical influence, these findings suggest that pediatric FFT does not necessarily produce detectable changes in global walking performance during self-selected gait. Previous studies have shown that proximal joint kinematics and inter-articular coordination may be redistributed when peripheral muscle function or arch support mechanisms are altered [22–24]. The present kinematic findings are compatible with such an adaptive response; however, because muscle activation and joint kinetics were not measured, the underlying neuromuscular control strategy cannot be determined directly from the current data. Therefore, preservation of macroscopic gait should be interpreted as a functional observation rather than as evidence of a specific central compensatory mechanism.

The static analysis provides further evidence that the biomechanical influence of FFT may extend beyond the foot and ankle. During standing, the affected hip showed a significant shift toward relative internal rotation (−2.11°) compared with the unaffected limb (−5.79°). Souza et al. [25] previously reported temporal coupling between hindfoot pronation and hip internal rotation during gait. In this context, the present findings extend the observation of distal–proximal coupling to static alignment and suggest that altered foot posture may coexist with changes in proximal joint orientation even before dynamic gait demands are considered. Importantly, the absolute angular difference observed in this study was modest. It should therefore not be interpreted in isolation as evidence of pathological hip dysfunction or as an indication for intervention. Rather, it may represent a subtle alteration in lower-extremity alignment that warrants consideration together with dynamic gait findings.

During walking, the affected limb demonstrated a significantly increased Hip Abduction/Adduction GVS (*P =* 0.032), indicating greater deviation in frontal-plane hip kinematics. Static structural malalignment has previously been associated with dynamic functional alterations [10], while femoral internal rotation and hip adduction have been biomechanically linked to dynamic knee valgus patterns [26,27]. Hip abductors such as the gluteus medius play an important role in frontal-plane pelvic and hip stability during single-limb support [28]. Accordingly, the combination of altered static hip orientation and increased frontal-plane GVS observed in this study provides evidence of a localized proximal kinematic alteration associated with FFT. However, GVS quantifies kinematic deviation and does not directly measure muscle activation, joint loading, or neuromuscular effort. Therefore, the present findings cannot establish that greater hip-abductor activation or increased mechanical loading actually occurred.

The relatively small magnitude of the observed angular changes also requires careful clinical interpretation. In growing children, a statistically detectable difference does not necessarily represent clinically important dysfunction, particularly when conventional spatiotemporal gait parameters remain unaffected. Nevertheless, the coexistence of altered static hip alignment and increased dynamic hip kinematic deviation suggests that localized biomechanical changes may be detectable before obvious deterioration in gross walking performance becomes apparent. The clinical implication is therefore not that children with FFT require intervention solely on the basis of these small angular differences, but that assessment limited to foot morphology or global gait parameters may overlook proximal movement characteristics. Incorporating static lower-extremity alignment and hip kinematic assessment may provide complementary information when evaluating children with FFT, particularly during follow-up.

Despite altered static hip alignment and elevated dynamic frontal-plane GVS, the absence of significant differences in macroscopic spatiotemporal parameters is consistent with the substantial adaptability of pediatric locomotion described previously [29]. However, the present cross-sectional kinematic data cannot determine whether the observed pattern represents an active neuromuscular compensatory strategy, nor can they establish whether these alterations persist, progress, or contribute to future musculoskeletal injury. Previous studies have associated persistent lower-extremity malalignment and altered joint mechanics with cumulative musculoskeletal loading and degenerative changes [13,30,31], providing a rationale for further investigation. These studies should not, however, be taken as evidence that the children examined here are at increased long-term injury or degenerative risk. Longitudinal follow-up incorporating kinetic and EMG measurements will be necessary to determine the functional consequences and prognostic relevance of the kinematic differences observed in the present study.

Overall, the value of the present study lies less in demonstrating gross gait dysfunction than in identifying a pattern in which pediatric FFT is associated with selective proximal alignment and kinematic differences despite preserved macroscopic walking characteristics. This provides a complementary perspective to previous studies of flatfoot biomechanics by jointly considering static alignment, dynamic gait, and affected–unaffected limb differences within the same analytical framework. Clinically, these findings support a broader kinetic-chain assessment of pediatric FFT while emphasizing that small kinematic deviations should be interpreted cautiously and in conjunction with symptoms, function, and longitudinal changes.

### Limitations

First, due to its cross-sectional design, this study can only confirm an association between arch collapse and proximal joint malalignment, precluding the establishment of causality. Future longitudinal studies are warranted to elucidate how structural changes in the foot dynamically influence the developmental trajectory of proximal joints. Second, because pediatric FFT is typically bilateral, the unilateral group was relatively small. We therefore did not perform age-stratified analyses to avoid further reducing subgroup sizes and statistical reliability. Although linear mixed-effects models are robust to unbalanced designs, this sample discrepancy may still limit statistical power and mask age-specific differences. Future studies with larger, more balanced cohorts are needed to validate these findings. Third, this investigation primarily relied on kinematic parameters, lacking concurrent kinetic and electromyographic (EMG) data. This limits our ability to directly quantify joint loading, energy expenditure, and specific muscular compensatory strategies. Integrating synchronized force platforms with surface EMG will be crucial for elucidating the precise biomechanical costs in future studies. Finally, skin-marker-based optical motion capture is inherently susceptible to soft tissue artifacts (STAs), particularly around the pelvis and hip regions, which may influence the accuracy of the kinematic data.

This study also had limitations inherent to its retrospective design. Data were collected by two experienced physicians using a standardized protocol, which reduced operator-related variability; however, potential confounders such as physical activity, footwear habits, and rehabilitation history could not be fully controlled. In addition, the absence of follow-up data precluded evaluation of longitudinal changes. Future prospective studies are needed to address these issues.

## Conclusion

This study showed that pediatric FFT was associated with selective proximal biomechanical alterations, including relative hip internal rotation during static stance and increased frontal-plane hip kinematic deviation during walking, despite largely preserved macroscopic spatiotemporal gait parameters. These findings suggest that the biomechanical influence of FFT may extend beyond the foot and ankle and may not be fully captured by conventional gait measures alone. However, the observed angular differences were modest, and the present kinematic data do not establish specific neuromuscular compensatory mechanisms or long-term injury risk. Static lower-extremity alignment and dynamic hip kinematics may therefore provide complementary information in the clinical assessment and follow-up of pediatric FFT. Longitudinal studies incorporating kinetic and EMG measurements are needed to determine the clinical and prognostic significance of these alterations.

## Supporting information

S1 Fig Comparisons of mean velocity, stride frequency and step width. Violin plots show group comparisons of mean velocity, stride frequency, and step width. No statistically significant differences were detected.
(TIF)

S2 Fig Comparisons of temporal‑spatial gait parameters and gait cycle. Bar charts illustrate temporal parameters, spatial parameters, and gait‑cycle comparisons. No statistically significant differences were found.
(TIF)

S3 Fig Comparisons of static joint angles among study groups. Bar charts represent static joint‑angle comparisons. Significant difference was observed in hip rotation angle (P < 0.05).
(TIF)

S4 Fig Joint‑level GPS, GDI and GVS comparisons. Group comparisons for GPS, GDI and GVS at each joint. Significant difference was found in hip abduction/adduction GVS (P < 0.05).
(TIF)

S1 Table Demographic characteristics of the participants. (DOCX)

S2 Table Comparison of gait parameters between unilateral flatfoot and bilateral flatfoot. (DOCX)

S3 Table Comparison of spatio-temporal parameters between normal foot and flat foot. (DOCX)

S4 Table Comparison of static joint angles between normal foot and flat foot. (DOCX)

S5 Table Comparison of gait variability between normal foot and flat foot. (DOCX)
