# Supplementary material for: Compensatory proximal malalignment despite preserved macroscopic gait in pediatric flexible flatfoot: A dual-level analysis of static alignment and dynamic kinematics
Source: PLoS One. 2026 Sep 16;21(9):e0358183. doi: 10.1371/journal.pone.0358183 (PMC13581023; doi:10.1371/journal.pone.0358183)
Supplement: Supplementary file 1 [file pone.0358183.s005.docx]

**Table 1. Demographic characteristics of the participants**

| Characteristics | UFFT (N=25) | BFFT(N=128) | Z/χ2 | *P* |
| --- | --- | --- | --- | --- |
| Age(year) | 12.00 (11.00, 14.00) | 12.00 (10.00, 15.00) | Z = -0.206 | 0.837 |
| Height(m) | 162.00 (151.50, 170.00) | 160.00 (150.00, 170.75) | Z = -0.417 | 0.677 |
| Weight(kg) | 49.00 (41.50, 61.75) | 50.00 (39.63, 65.00) | Z = -0.111 | 0.912 |
| BMI (kg/m^2^) | 18.25 (16.42, 22.95) | 20.05 (16.74, 23.34) | Z = -0.600 | 0.549 |
| Male/female | 15 (60.0%) / 10 (40.0%) | 74 (57.8%) / 54 (42.2%) | χ² = 0.041 | 0.839 |

UFFT: Unilateral flexible flatfoot; BFFT: Bilateral flexible flatfoot; m: meter; kg: kilogram
