# Supplementary material for: Compensatory proximal malalignment despite preserved macroscopic gait in pediatric flexible flatfoot: A dual-level analysis of static alignment and dynamic kinematics
Source: PLoS One. 2026 Sep 16;21(9):e0358183. doi: 10.1371/journal.pone.0358183 (PMC13581023; doi:10.1371/journal.pone.0358183)
Supplement: Supplementary file 2 [file pone.0358183.s006.docx]

**Table 2. Comparison of gait parameters between unilateral flatfoot and bilateral flatfoot**

| Parameters | UFFT (N=25) | BFFT (N=128) | Z | *P* |
| --- | --- | --- | --- | --- |
| Mean Velocity(m/s) | 1.00 (1.00, 1.10) | 1.00 (0.90, 1.10) | -0.384 | 0.701 |
| Frequency(steps/min) | 108.00(99.60,114.60) | 107.40(100.95,115.65) | -0.400 | 0.689 |
| Step Width(m) | 0.11 (0.08 0.14) | 0.11 (0.09, 0.14) | -0.812 | 0.417 |

UFFT:Unilateral flexible flatfoot; BFFT: Bilateral flexible flatfoot; steps/min: steps per minute; m/s: meter per second.
