# Supplementary material for: Compensatory proximal malalignment despite preserved macroscopic gait in pediatric flexible flatfoot: A dual-level analysis of static alignment and dynamic kinematics
Source: PLoS One. 2026 Sep 16;21(9):e0358183. doi: 10.1371/journal.pone.0358183 (PMC13581023; doi:10.1371/journal.pone.0358183)
Supplement: Supplementary file 3 [file pone.0358183.s007.docx]

**Table 3. Comparison of spatio-temporal parameters between normal foot and flat foot**

| Parameters | Normal(N=25) | FFT (N=281) | Mean Diff (95% CI) | *P* |
| --- | --- | --- | --- | --- |
| Stride Time (s) | 1.14 ± 0.03 | 1.13 ± 0.01 | 0.01 (-0.04, 0.06) | 0.614 |
| Stance Time (s) | 0.71 ± 0.02 | 0.71 ± 0.01 | 0.01 (-0.02, 0.04) | 0.602 |
| Swing Time (s) | 0.43 ± 0.02 | 0.43 ± 0.01 | 0.00 (-0.04, 0.04) | 0.989 |
| Stance Phase (%) | 62.07 ± 1.59 | 62.30 ± 0.54 | -0.23 (-3.45, 2.99) | 0.889 |
| Swing Phase (%) | 37.95 ± 1.32 | 37.74 ± 0.48 | 0.21 (-2.45, 2.87) | 0.876 |
| Single Support Phase (%) | 36.40 ± 1.46 | 37.96 ± 0.48 | -1.56 (-4.52, 1.40) | 0.301 |
| Double Support Phase (%) | 13.16 ± 2.53 | 15.00 ± 0.73 | -1.84 (-7.06, 3.38) | 0.489 |
| Stride Length (m) | 1.14 ± 0.02 | 1.14 ± 0.01 | 0.00 (-0.04, 0.05) | 0.844 |
| Step Length (m) | 0.58 ± 0.01 | 0.57 ± 0.01 | 0.01 (-0.01, 0.04) | 0.261 |

FFT: flexible flatfoot.s: second; m: meter; %: percentage.
