# Supplementary material for: Compensatory proximal malalignment despite preserved macroscopic gait in pediatric flexible flatfoot: A dual-level analysis of static alignment and dynamic kinematics
Source: PLoS One. 2026 Sep 16;21(9):e0358183. doi: 10.1371/journal.pone.0358183 (PMC13581023; doi:10.1371/journal.pone.0358183)
Supplement: Supplementary file 4 [file pone.0358183.s008.docx]

**Table 4 Comparison of static joint angles between normal foot and flat foot**

| Parameters | Normal(N=25) | FFT (N=281) | Mean Diff (95% CI) | *P* |
| --- | --- | --- | --- | --- |
| Pelvis Tilt(°) | 12.01 ± 1.16 | 11.71 ± 0.69 | 0.30 (-1.71, 2.32) | 0.767 |
| Pelvis Obliquity(°) | 0.59 ± 0.57 | -0.05 ± 0.17 | 0.64 (-0.54, 1.81) | 0.285 |
| Pelvis Rotation(°) | 0.25 ± 1.30 | -0.02 ± 0.38 | 0.28 (-2.39, 2.95) | 0.838 |
| Hip Flexion/Extension(°) | 6.09 ± 1.14 | 6.48 ± 0.88 | -0.39 (-1.95, 1.17) | 0.62 |
| Hip Abduction/Adduction(°) | -3.94 ± 0.88 | -3.18 ± 0.24 | -0.76 (-2.57, 1.05) | 0.411 |
| Hip Rotation(°) | -5.79 ± 1.79 | -2.11 ± 0.67 | -3.68 (-7.25, -0.12) | **0.043^*^** |
| Knee Flexion/Extension(°) | 1.29 ± 0.93 | 1.83 ± 0.49 | -0.54 (-2.26, 1.18) | 0.535 |
| Ankle Dorsi/Plantarflexion(°) | 4.40 ± 1.35 | 2.80 ± 0.60 | 1.60 (-1.01, 4.21) | 0.228 |
| Foot Progression Angle(°) | -14.29 ± 1.88 | -13.65 ± 0.62 | -0.64 (-4.46, 3.17) | 0.74 |

FFT: flexible flatfoot; °: degree.
