# Supplementary material for: Compensatory proximal malalignment despite preserved macroscopic gait in pediatric flexible flatfoot: A dual-level analysis of static alignment and dynamic kinematics
Source: PLoS One. 2026 Sep 16;21(9):e0358183. doi: 10.1371/journal.pone.0358183 (PMC13581023; doi:10.1371/journal.pone.0358183)
Supplement: Supplementary file 5 [file pone.0358183.s009.docx]

**Table 5.Comparison of gait variability between normal foot and flat foot**

| Parameters | Normal(N=25) | FFT (N=281) | Mean Diff (95% CI) | *P* |
| --- | --- | --- | --- | --- |
| GDI（Sc） | 86.02 ± 1.54 | 85.91 ± 0.71 | 0.11 (-2.83, 3.05) | 0.943 |
| GPS(°) | 10.37 ± 0.44 | 10.55 ± 0.21 | -0.18 (-1.02, 0.67) | 0.679 |
| Pelvis Tilt GVS(°) | 6.70 ± 0.49 | 6.80 ± 0.46 | -0.10 (-0.47, 0.27) | 0.588 |
| Pelvis Obliquity GVS(°) | 3.63 ± 0.37 | 3.52 ± 0.14 | 0.12 (-0.62, 0.85) | 0.754 |
| Pelvis Rotation GVS(°) | 4.96 ± 0.39 | 4.74 ± 0.18 | 0.22 (-0.54, 0.98) | 0.567 |
| Hip Flexion/Extension GVS(°) | 13.06 ± 0.90 | 13.56 ± 0.58 | -0.50 (-2.01, 1.00) | 0.509 |
| Hip Abduction/Adduction GVS(°) | 4.45 ± 0.43 | 5.40 ± 0.14 | -0.95 (-1.82, -0.08) | **0.032^*^** |
| Hip Rotation GVS(°) | 10.99 ± 1.02 | 12.31 ± 0.33 | -1.32 (-3.41, 0.76) | 0.213 |
| Knee Flexion/Extension GVS(°) | 15.13 ± 1.05 | 14.97 ± 0.38 | 0.17 (-1.94, 2.27) | 0.877 |
| Ankle Dorsi/Plantarflexion GVS(°) | 11.42 ± 0.98 | 10.98 ± 0.37 | 0.44 (-1.51, 2.39) | 0.654 |
| Foot Progression GVS(°) | 9.74 ± 0.81 | 8.51 ± 0.29 | 1.22 (-0.40, 2.85) | 0.14 |

FFT: flexible flatfoot.; GDI: Gait Deviation Index; GPS: Gait Profile Score; GVS: Gait Variable Score; °: degree; Sc: score.
